# Reference right atrial dimensions and volume estimation by steady state free precession cardiovascular magnetic resonance

**DOI:** 10.1186/1532-429X-15-29

**Published:** 2013-04-08

**Authors:** Alicia M Maceira, Juan Cosín-Sales, Michael Roughton, Sanjay K Prasad, Dudley J Pennell

**Affiliations:** 1 Cardiac Imaging Unit, ERESA, Valencia, Spain; 2 Department of Cardiology, Hospital Arnau de Vilanova, Valencia, Spain; 3 Medical Statistics Department, Royal Brompton Hospital, London, UK; 4 Cardiovascular Magnetic Resonance Unit, Royal Brompton Hospital, Sydney Street, London SW3 6NP, United Kingdom

**Keywords:** Magnetic resonance, Heart, Right atrial volume, Dimensions, Reference values

## Abstract

**Background:**

Cardiovascular magnetic resonance (CMR) steady state free precession (SSFP) cine sequences with high temporal resolution and improved post-processing can accurately measure RA dimensions. We used this technique to define ranges for normal RA volumes and dimensions normalized, when necessary, to the influence of gender, body surface area (BSA) and age, and also to define the best 2D images-derived predictors of RA enlargement.

**Methods:**

For definition of normal ranges of RA volume we studied 120 healthy subjects (60 men, 60 women; 20 subjects per age decile from 20 to 80 years), after careful exclusion of cardiovascular abnormality. We also studied 120 patients (60 men, 60 women; age range 20 to 80 years) with a clinical indication for CMR in order to define the best 1D and 2D predictors of RA enlargement. Data were generated from SSFP cine CMR, with 3-dimensional modeling, including tracking of the atrioventricular ring motion and time-volume curves analysis.

**Results:**

In the group of healthy individuals, age influenced RA 2-chamber area and transverse diameter. Gender influenced most absolute RA dimensions and volume. Interestingly, right atrial volumes did not change with age and gender when indexed to body surface area. New CMR normal ranges for RA dimensions were modeled and displayed for clinical use with normalization for BSA and gender and display of parameter variation with age. Finally, the best 2D images-derived independent predictors of RA enlargement were indexed area and indexed longitudinal diameter in the 2-chamber view.

**Conclusion:**

Reference RA dimensions and predictors of RA enlargement are provided using state-of-the-art CMR techniques.

## Background

Right atrial (RA) enlargement may occur in numerous conditions including congenital heart disease, acquired valvular disease, pulmonary disorders, and heart failure. Remodeling of the RA has also been reported in patients with paroxysmal atrial fibrillation (AF) [1-3], and recent data have shown that both left atrial (LA) and RA remodeling are equally associated with recurrence of AF after cardioversion [4]. Interestingly, RA and LA remodeling may coexist because one predisposes the heart to the other, so their combined enlargement may better express more remarkable structural remodeling and, more importantly, the combination of these conditions might be a better prognostic indicator of AF recurrence than either alone. Another potential clinical value of RA measurement, since RA size is at least partially determined by the same factors that affect diastolic right ventricular (RV) filling, is its ability to act as an early marker of RV dysfunction, which often precedes systolic dysfunction in a variety of conditions affecting the RV. Furthermore, it may provide significant prognostic information in patients with chronic systolic heart failure [5] and pulmonary hypertension [6,7].

Atrial remodeling is most accurately estimated by measuring atrial volume, but in current clinical practice this is not routinely performed. Cardiovascular magnetic resonance (CMR) is the gold standard technique for measurement of ventricular dimensions and function with reference ranges established from the Steady State Free Precession (SSFP) technique [8,9], and this has also been reported for the left atrium [10]. Some studies on RA reference values have been published [11-13], but RA dimensions have not been extensively studied with CMR as well as the systematic analysis of the influences of age, gender and body surface area (BSA). Therefore, the aim of this study was to establish SSFP based reference values in normal subjects for RA dimensions normalized for independent influences such as gender, body surface area and age when required. We also aimed to determine the best predictors of right atrial enlargement among 1D and 2D parameters, and relate RA volume to RA diameters and areas, as these are easily obtained in the clinical setting.

## Methods

### Normals and patients

For definition of normal ranges of RA dimensions we studied 120 subjects, with 10 men and 10 women in each of 6 age deciles from 20 to 80 years. This cohort of healthy subjects has been used for defining left and right ventricular and LA reference dimensions. Their baseline characteristics have been published previously [8]. In brief, all subjects were normotensives (hypertension defined as systolic blood pressure ≥ 140 mmHg and/or diastolic blood pressure ≥ 90 mmHg), asymptomatic, with no known risk factors or history of cardiac disease, and with normal physical examination and electrocardiogram (ECG). Height, weight, blood pressure, total cholesterol, HDL and B-natriuretic peptide were measured in all. BSA was calculated according to the Mosteller formula [14]. The coronary artery disease risk over 10 years was calculated [15]. BNP levels were 2.5 ±2.1 pg/mL (range 0.5 – 12.0), and all were in the normal range (<100 pg/mL) [16]. Therefore, as far as it was possible to ascertain with conventional noninvasive techniques, all the apparently healthy volunteers had a normal cardiovascular system with no high blood pressure and no evidence of heart failure. In a second step, and in order to define the best 1D and 2D predictors of RA enlargement, a group of 120 patients (60 men and 60 women, age range 20–80) that were referred to CMR for clinical reasons, who were in sinus rhythm and who agreed to participate in the study, were included. The main reasons for referral to CMR have been published elsewhere [10] and are briefly summarized in Table 1. Research was in compliance with the Helsinki Declaration. The study was approved by the institutional Ethics Committee of the Royal Brompton Hospital, and all subjects gave written informed consent.

**Table 1:** Baseline characteristics of the healthy subjects and the patient group (mean ± SD)

	**Healthy subjects**	**Patients**
N	120	120
Males	50%	53%
Age [yr] (min, max)	49 ± 17 (20, 80)	65 ± 12 (20, 80)
Height [cm]	171 ± 9	163 ± 9
Weight [kg]	72 ± 13	76 ± 13
Body surface area [m^2^]	1.83 ± 0.18	1.82 ± 0.18
Body mass index [kg/m^2^]	24 ± 4	29 ± 5
Heart Rate [bpm]	66 ± 10	69 ± 13
Systolic blood pressure [mmHg]	124 ± 12	140 ± 25
Diastolic blood pressure [mmHg]	73 ± 7	77 ± 14
Pathology (n)		
Ischemic heart disease	-	46
Coronary risk factors	-	35
Hypertensive heart disease	-	12
Valvular heart disease	-	13
Dilated cardiomyopathy	-	4
Restrictive cardiomyopathy	-	2
Congenital heart disease	-	3
Myocarditis	-	2
Hypertrophic cardiomyopathy	-	1
Arrhythmogenic right ventricular cardiomyopathy	-	1
Pericardial disease	-	1

### CMR

CMR was performed with 1.5 T scanners (Siemens Sonata and Avanto) using front and back surface coils and retrospective ECG triggering for capture of the entire cardiac cycle including diastole. All CMR scans were performed by the same operator. SSFP end-expiratory breath-hold cines were acquired in the two (left and right chambers) and four chamber views, with subsequent contiguous short-axis cines from the atrioventricular (AV) ring to the base of the atria. Slice thickness was 5 mm with no gap between slices. The temporal resolution was 21 ±1 ms. Sequence parameters included repetition time/echo time of 3.2/1.6 ms, in-plane pixel size of 2.1 × 1.3 mm, flip angle 60º, and acquisition time of typically 18 heartbeats.

### CMR analysis

**Figure 1:**
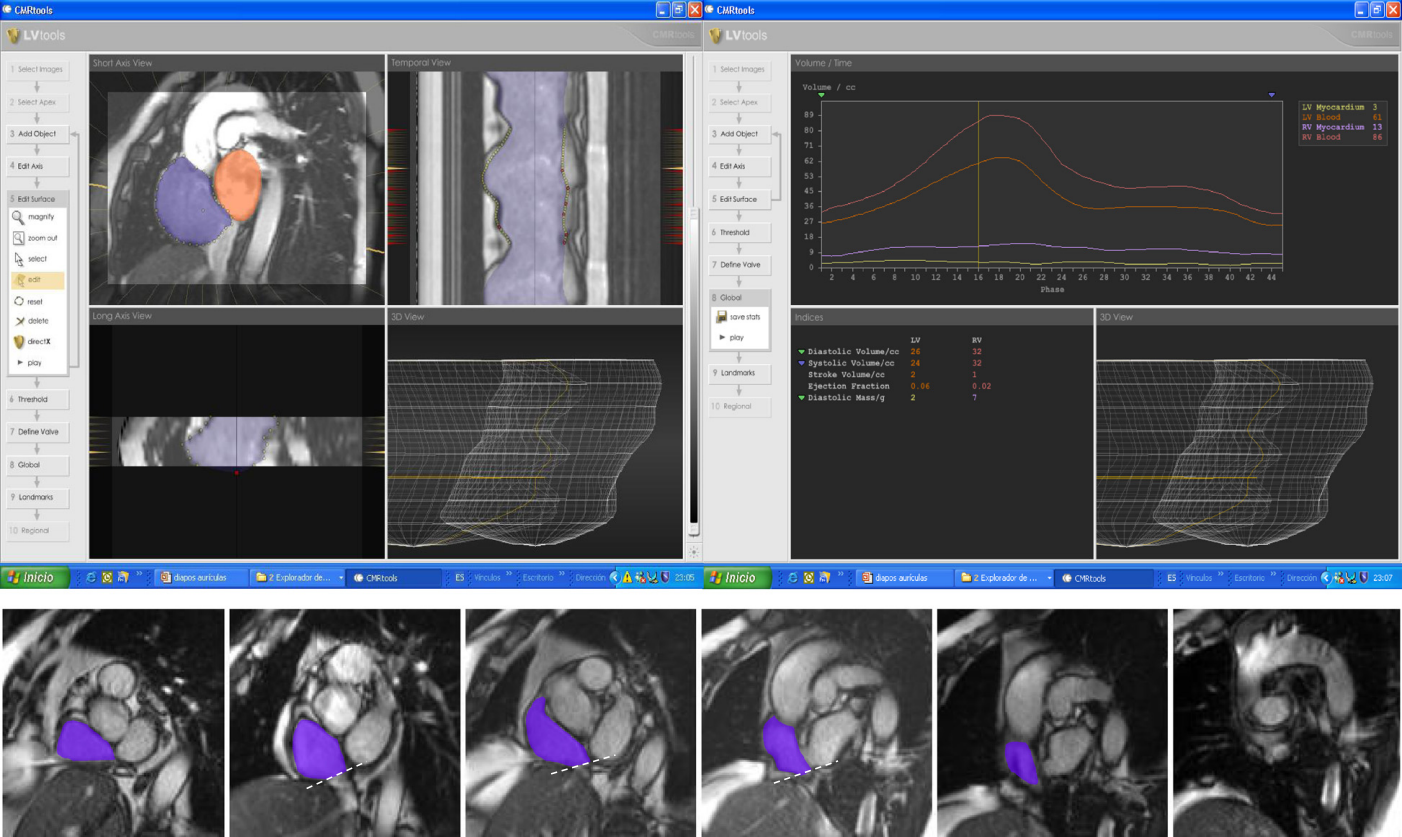
**CMR analysis of atrial volumes.** Right atrial endocardial borders were delineated in all planes in all cardiac phases with inclusion of the atrial appendage and exclusion of the cava veins. The systolic descent and twist of the tricuspid valve was calculated from tracking of the valve motion on the long axis cines. The phase at which the atrial volume is at a maximum was selected for quantification.

**Figure 2:**
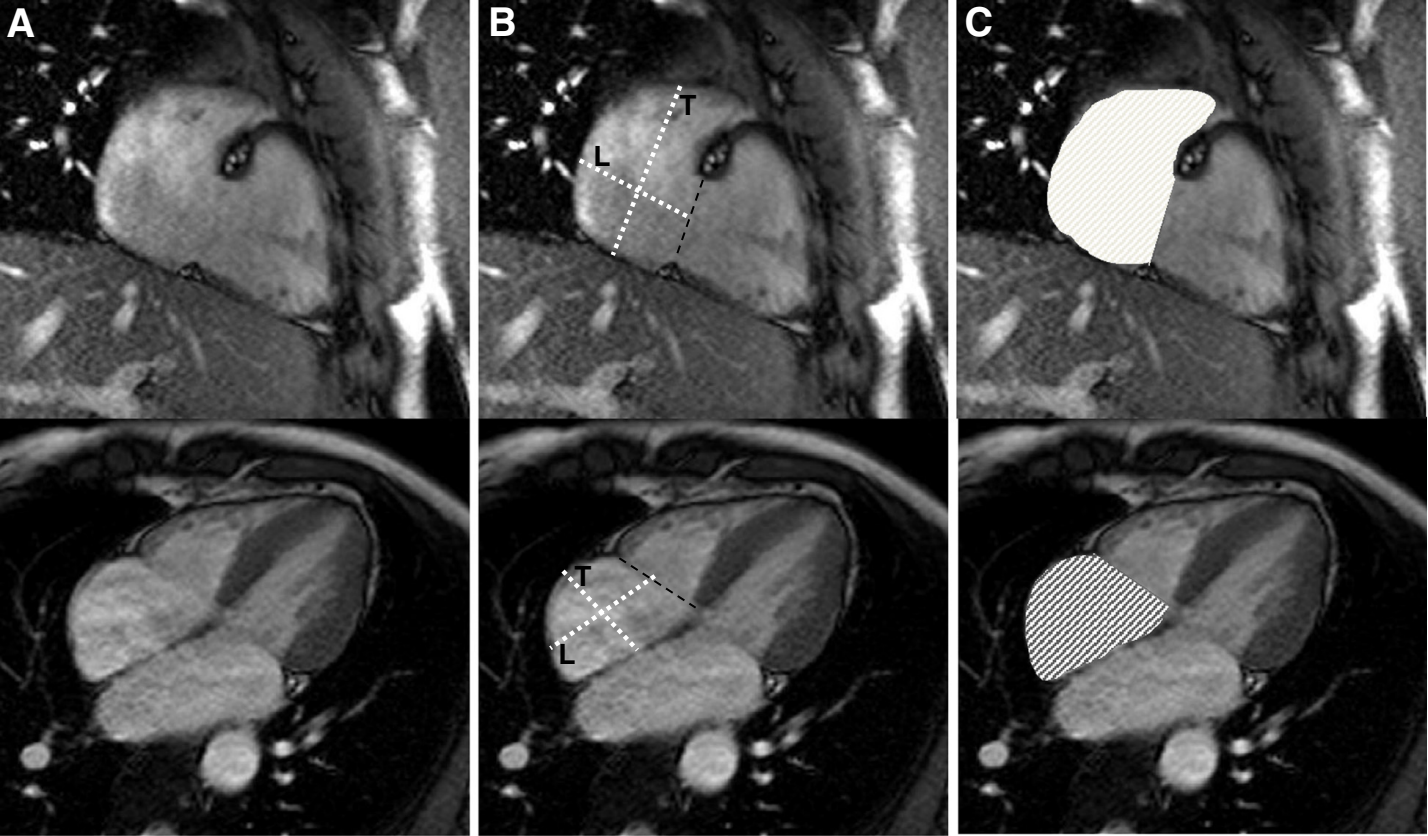
**Measurement of right atrial parameters.** Areas and diameters were measured in the phase of the cardiac cycle at which the atrial size was at a maximum. The figure shows the 2-chamber (top) and 4-chamber (bottom) views in which measurements were done. In **B**), longitudinal diameter (L) is obtained from the posterior wall of the right atrium to the center of the tricuspid plane, and transverse diameter (T) is obtained perpendicular to the longitudinal diameter, at the mid level of the right atrium. In **C**) measured areas are shown for both views.

Analysis was performed with a personal computer and semi-automated software (CMRtools, Cardiovascular Imaging Solutions, London, UK). In all subjects (healthy controls and patients) RA maximum volume was measured as well as maximum diameters and areas, measured in the 42-chamber and right 2-chamber views. Atrial volume analysis included 2 steps: First, delineation of the atrial endocardial border in all planes in all cardiac phases. Second, the systolic descent and twist of the tricuspid valve was calculated from tracking of the valve motion on the long axis cines, and used to correct for increase in atrial volume due to AV ring descent (Figure 1). We included the atrial appendage and excluded the cava veins. Other approaches are possible, but the arguments for and against are not decisive. All diameters and areas derived from 2D images were measured in the phase of the corresponding cine sequences at which the atrial size and volume measurements were at a maximum. The longitudinal diameter was measured form the midpoint of the line between the lateral and septal (or superior and inferior in the 2-chamber view) insertion of the tricuspid valve to the roof of the right atrium. Transverse diameter was measured perpendicular to the midpoint of the longitudinal diameter (Figure 2).

### Statistical analysis

This was carried out with the statistical software SPSS Statistics 17.0 (IBM, United States). All atrial parameters were found to satisfy a normal distribution using the Kolmogorov-Smirnov test and summary data for these variables are presented as mean ± SD. Intra and interobserver reproducibility were tested in 50 subjects belonging to the cohort of healthy subjects. Simple linear regression was used to model the indexed data and to construct reference ranges as mean and 95% confidence intervals. Multivariable analysis was used to analyze variations in parameters due to age and gender. P values <0.05 were considered significant. In the patient group, correlations of 1D and 2D parameters with RA volume were assessed with the Pearson’s coefficient. Logistic regression analysis was used to define the best predictors of RA enlargement among 1D and 2D parameters. Linear regression analysis was used to develop a method to predict RA volume with these parameters.

## Results

### Baseline characteristics and summary results for the healthy subjects group

Table 1 summarizes the baseline characteristics of the healthy subjects included for defining normal reference values. The reproducibility study was undertaken by 2 operators with more than 5 years’ experience in CMR. One operator assessed intraobserver variability and the other one, blinded to previous results, interobserver variability. Intraobserver variability [17] was 3.5% for RA volume, 3.6% and 1.8% for areas in the 2-chamber and 4-chamber views, 4.1% and 4.2% for longitudinal and transverse diameters in the 2-chamber view, and 4.1% and 4% for longitudinal and transverse diameters in the 4-chamber view, respectively. Interobserver variability was 3.9% for RA volume, 5.2% and 5% for areas in the 2-chamber and 4-chamber views, 5.5% and 5.5% for longitudinal and transverse diameters in the 2-chamber view, and 5.8% and 5.1% for longitudinal and transverse diameters in the 4-chamber view, respectively. RA reference values with differentiation into males, females and all subjects, without age breakdown, and sub-division into absolute and body surface area normalized values are shown in Table 2, which have application in studies of unsorted individuals. Parameters that showed differences with age are also depicted, with age breakdown, in Table 3.

**Table 2:** Healthy subjects- Right atrial summary data for all ages (mean ± SD, 95% confidence interval)

	**All**	**Males**	**Females**
Volume [mL] SD 20	100 (61, 139)	109 (64, 154)	91 (58, 124)
Volume/BSA [mL/m^2^] SD 10.3	54 (34, 75)	55 (33, 78)	53 (36, 70)
Area – 4 ch [cm^2^] SD 3.8	22 (15, 30)	24 (15, 33)	20 (15, 26)
Area/BSA – 4 ch [cm^2^/m^2^] SD 1.8	12 (8, 15)	12 (8, 16)	12 (9, 15)
Longitudinal diameter – 4 ch [cm] SD 0.58	5.5 (4.3, 6.6)	5.6 (4.6, 6.7)	5.3 (4.3, 6.4)
Longitudinal diameter/BSA – 4 ch [cm/m^2^] SD 0.32	3.0 (2.4, 3.6)	2.9 (2.5, 3.3)	3.1 (2.6, 3.6)
Transverse diameter – 4 ch [cm] SD 0.55	4.7 (3.7, 5.8)	5.0 (3.7, 6.4)	4.5 (3.6, 5.4)
Transverse diameter/BSA – 4 ch [cm/m^2^] SD 0.3	2.6 (2.0, 3.2)	2.6 (1.9, 3.3)	2.6 (2.2, 3.1)
Area – 2 ch [cm^2^] SD 3.95*	22 (14, 29)	23 (14, 31)	21 (14, 27)
Area/BSA – 2 ch [cm^2^/m^2^] SD 2.27 *	12 (7, 16)	12 (7, 16)	12 (8, 16)
Longitudinal diameter – 2 ch [cm] SD 0.5	5.4 (4.4, 6.5)	5.7 (4.6, 6.8)	5.1 (4.1, 6.1)
Longitudinal diameter/BSA – 2 ch [cm/m^2^] SD 0.3	2.9 (2.4, 3.5)	2.9 (2.3, 3.4)	3.0 (2.0, 4.0)
Transverse diameter – 2 ch [cm] SD 0.7 *	4.3 (3.0, 5.7)	4.3 (3.1, 5.5)	4.4 (3.3, 5.5)
Transverse diameter/BSA – 2 ch [cm/m^2^] SD 0.4 *	2.4 (1.5, 3.2)	2.2 (1.5, 2.9)	2.6 (1.5, 3.7)

*BSA* – body surface area; *4 ch* – 4-chamber view; *2 ch* – 2-chamber view.

* Significant differences (p < 0.05) among age groups on multivariate analysis.

**Table 3:** Healthy subjects- Right atrial parameters with significant differences with age on multivariate analysis (mean, 95% confidence interval)

	**20-29 years**	**30-39 years**	**40-49 years**	**50-59 years**	**60-69 years**	**70-79 years**
	**All subjects**					
RA area-2 ch [cm^2^] SD 4.5	24 (16, 32)	23 (15, 31)	22 (14, 30)	21 (13, 29)	20 (12, 28)	19 (11, 27)
RA area/BSA- 2 ch [cm^2^/m^2^] SD 2.5	13 (9, 18)	13 (8, 17)	12 (8, 17)	12 (7, 16)	11 (6, 15)	10 (6, 15)
RA transverse diameter- 2 ch [cm] SD 0.6	5.1 (3.7, 6.4)	4.7 (3.4, 6.1)	4.4 (3.1, 5.8)	4.1 (2.8, 5.5)	3.8 (2.5, 5.2)	3.5 (2.1, 4.8)
RA transverse diameter/BSA- 2 ch [cm/m^2^] SD 0.4	2.8 (2.1, 3.6)	2.7 (1.8, 3.5)	2.5 (1.6, 3.4)	2.3 (1.4, 3.2)	2.2 (1.3, 3.1)	2.0 (1.1, 2.9)
	**Males**					
RA area-2 ch [cm^2^] SD 4.5	25 (16, 33)	24 (15, 33)	23 (14, 32)	22 (14, 31)	21 (13, 30)	21 (12, 29)
RA area/BSA- 2 ch [cm^2^/m^2^] SD 2.5	13 (8, 17)	12 (8, 17)	12 (7, 17)	11 (7, 16)	11 (6, 16)	10 (6, 15)
RA transverse diameter- 2 ch [cm] SD 0.6	4.9 (3.7, 6.2)	4.7 (3.5, 6.0)	4.5 (3.3, 5.7)	4.2 (3.0, 5.4)	4.0 (2.8, 5.2)	3.7 (2.5, 4.9)
RA transverse diameter/BSA- 2 ch [cm/m^2^] SD 0.4	2.5 (1.8, 3.2)	2.4 (1.7, 3.1)	2.3 (1.6, 3.0)	2.1 (1.4, 2.8)	2.0 (1.3, 2.7)	1.9 (1.2, 2.6)
	**Females**					
RA area-2 ch [cm^2^] SD 3.4	24 (17, 31)	23 (16, 29)	21 (15, 28)	20 (13, 27)	19 (12, 25)	18 (11, 24)
RA area/BSA- 2 ch view [cm^2^/m^2^] SD 2	14 (10, 18)	13 (9, 18)	13 (8, 17)	12 (8, 16)	11 (7, 15)	10 (6, 14)
RA transverse diameter-2 ch [cm] SD 0.6	5.1 (4.0, 6.2)	4.8 (3.7, 5.9)	4.5 (3.4, 5.6)	4.2 (3.1, 5.3)	3.9 (2.8, 5.0)	3.6 (2.5, 4.7)
RA transverse diameter/BSA- 2 ch [cm/m^2^] SD 0.4	3.1 (2.3, 3.9)	2.9 (1.8, 4.0)	2.7 (1.6, 3.8)	2.5 (1.4, 3.6)	2.3 (1.2, 3.4)	2.1 (1.0, 3.2)

*LA* – left atrium; *AP* – anteroposterior; 2ch – 2-chamber view; *RA* – right atrium; *BSA* – body surface area; *SD* – standard deviation.

### Influence of body surface area on atrial parameters

BSA was significantly higher in males than in females (p <0.001). On multivariable analysis, BSA was found to have significant independent influence on all RA parameters except on 2-chamber area, and on transverse diameter in the 2-chamber view.

### Influence of age on atrial parameters

No significant increase in RA volume with age was observed in either univariable or multivariable analysis. On univariable analysis there was a significant decrease in absolute and normalized areas and transverse diameters (measured in the 2-chamber view) in males (p < 0.001), and in absolute and normalized areas in the 2-chamber view and normalized diameter in the 4-chamber view in females (p = 0.013, 0.046 and 0.043 respectively). On multivariable analysis, age was an independent predictor of absolute and normalized transverse diameters and areas (measured in the 2-chamber view) (all p = 0.001). Variables with significant differences according to age are depicted in Table 3.

### Influence of gender on atrial parameters

All absolute right atrial volume, diameters and areas were significantly larger in males (all p < 0.05) except transverse diameter in the 2-chamber view. When these parameters were normalized to BSA, only longitudinal (4-chamber view), and transverse (2-chamber view) diameters showed differences, being both higher in females. On multivariable analysis, gender had no significant independent influence on any variable.

### Predictors of atrial enlargement in the patient group

The baseline characteristics of the patient group are also depicted in Table 1. In this group of patients RA volumes showed a significant dispersion and, consequently, RA volume index (RAVi) ranged from 18 to 253 mL/m^2^ (mean ±SD 59 ±35 mL/m^2^). According to our own normal reference values reported in Table 2, 22 patients (11 males, 11 females) had left atrial enlargement (RAVi >78 mL/m^2^ in males and >70 mL/m^2^ in females). We aimed to determine the best independent predictors of RA enlargement, for which multivariate logistic regression analysis with forward selection procedure was performed for indexed 1D and 2D parameters. For the sake of simplicity, these parameters were included as categorical dichotomous variables (below or above each parameter’s upper limit of normal for all subjects). This analysis showed that the best predictors of RA enlargement were indexed area and indexed longitudinal diameter in the 2-chamber (Table 4).

**Table 4:** Predictors of right atrial enlargement according to RA volume index

**Univariate analysis:**	**OR**	**95%CI**	**p value**	**Chi square**
4-chamber longitudinal diameter (>6.6 cm)	13.6	6.97, 26.62	<0.001	NA
4-chamber transverse diameter (>5.8 cm)	5.0	2.41, 10.37	<0.001	18.7
4-chamber area (>30 cm2)	17.05	6.99, 41.17	<0.001	39.2
2-chamber longitudinal diameter (>6.5 cm)	9.6	4.14, 22.36	<0.001	27.7
2-chamber transverse diameter (>5.7 cm)	12.1	4.89, 30.12	<0.001	29
2-chamber area (>29 cm2)	21.6	6.83, 68.14	<0.001	27.4
4-chamber longitudinal diameter indexed (>3.6 cm/m2)	8.4	4.14, 17.12	<0.001	34.5
4-chamber transverse diameter indexed (>3.2 cm/m2)	8.4	3.29, 21.45	<0.001	19.8
4-chamber area indexed (>15 cm2/m2)	10.8	4.66, 25.18	<0.001	30.7
2-chamber longitudinal diameter indexed (>3.5 cm/m2)	12.0	3.71, 38.82	<0.001	17.2
2-chamber transverse diameter indexed (>3.2 cm/m2)	8.7	3.67, 20.44	<0.001	24.3
2-chamber area indexed (>16 cm2/m2)	38.5	9.55, 154.82	<0.001	26.4
**Multivariate analysis (indexed parameters):**	**OR**	**95%CI**	**p value**	**Chi square**
2-chamber area indexed (>16 cm2/m2)	6.71	2.35, 19.13	<0.001	32.9
2-chamber longitudinal diameter indexed (>3.5 cm/m2)	7.78	2.14, 28.35	0.002	

The upper limit of normal is shown in brackets according to Table 2.

### Estimation of right atrial volume from 2D based dimensions in the patient group

All 1D and 2D parameters correlated significantly with RA volume. The best correlations were found for areas measured in the 2-chamber (r = 0.904, p < 0.001) and 4-chamber views (r = 0.868, p < 0.001) (Table 5). Finally, linear regression analysis was used in the patient group in order to estimate RA volume (Table 6). All non-indexed 1D and 2D measurements were included in multiple linear regression analysis and the equation obtained was:

**Table 5:** Correlations of 1D and 2D parameters with RA volume (non-indexed parameters)

**Univariate analysis:**	**Pearson’s coefficient**	**p value**
4-chamber longitudinal diameter	0.840	<0.001
4-chamber transverse diameter	0.654	<0.001
4-chamber area	0.868	<0.001
2-chamber longitudinal diameter	0.746	<0.001
2-chamber transverse diameter	0.773	<0.001
2-chamber area	0.904	<0.001

**Table 6:** Predictors of right atrial volumes

**Univariate analysis:**	**Coeff**	**95%CI**	**p value**	**r squared**
4-chamber longitudinal diameter (cm)	48.4	42.7,54.1	<0.001	0.703
4-chamber transverse diameter (cm)	49.0	38.7,59.3	<0.001	0.428
4-chamber area (cm2)	6.72	6.0, 7.4	<0.001	0.752
2-chamber longitudinal diameter (cm)	42.7	35.1,50.3	<0.001	0.556
2-chamber transverse diameter (cm)	31.9	26.5,37.4	<0.001	0.573
2-chamber area (cm2)	4.6	4.2, 5.1	<0.001	0.806
4-chamber longitudinal diameter indexed (cm/m2)	63.7	52.2, 75.1	<0.001	0.508
4-chamber transverse diameter indexed (cm/m2)	77.1	58.6, 95.7	<0.001	0.365
4-chamber area indexed (cm2/m2)	11.1	9.7, 12.4	<0.001	0.698
2-chamber longitudinal diameter indexed (cm/m2)	66.4	52.4, 80.4	<0.001	0.470
2-chamber transverse diameter indexed (cm/m2)	51.4	41.4, 61.3	<0.001	0.512
2-chamber area indexed (cm2/m2)	7.8	7.0, 8.6	<0.001	0.772
**Multivariate analysis: non-indexed measurements**	**Coeff**	**95%CI**	**p value**	**r squared**
2-chamber area (cm2)	3.1	2.6, 3.6	<0.001	0.895
4-chamber area (cm2)	3.4	2.6, 4.1	<0.001	
Constant	−44.4			

**Figure 3:**
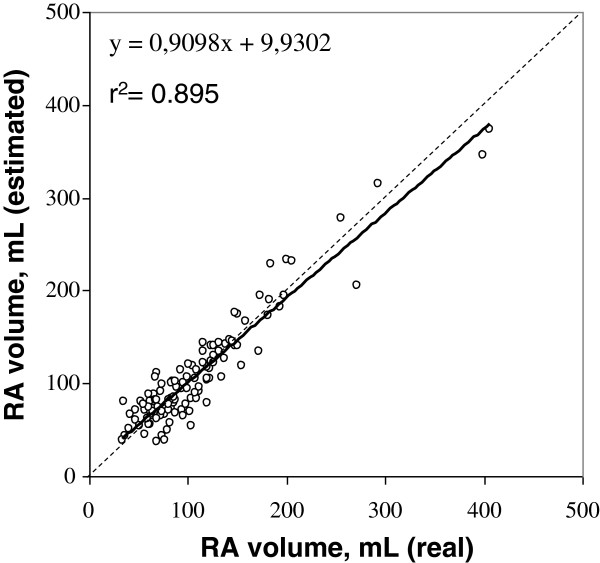
**Scatterplot showing the correlation between real RA volume and volume estimated with our method.** The dotted line is the line of identity and the solid line is the line of regression.

RA volume = 3.08*(2C area) +3.36*(4C area) -44.4 with an R^2^ of 0.895 (Figure 3).

**Figure 4:**
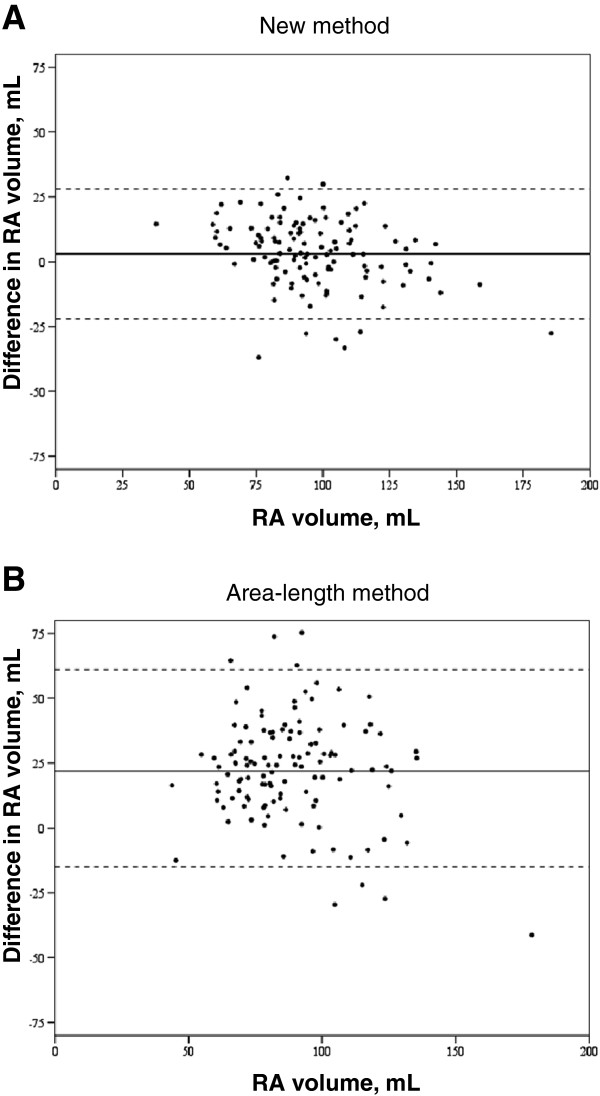
**Bland-Altman plot.** The continuous line represents the mean (bias) and the dotted lines represent the limits of agreement for **A**) our method of RA volume estimation and **B**) the area-length method, tested in the cohort of healthy subjects.

This method was then tested in the group of healthy subjects, and compared to the area-length method [18]. Correlation coefficient with real RA volume was r = 0.824 for our method and 0.649 for the area-length method. Both methods were shown to underestimate real volume, with a mean difference of 3.38 ± 13 mL for our method and 22.7 ± 19.8 mL for the area-length method (Figure 4).

## Discussion

Measurement of RA volume is not routinely performed clinically although its prognostic value has been shown in a number of conditions, such as chronic heart failure [5], atrial fibrillation [19] of pulmonary arterial hypertension [20]. This current study provides normal reference ranges for RA dimensions using state-of-the-art CMR acquisition techniques and analysis in a healthy moderately large population, which has been very well characterized for the absence of heart failure or any cardiomyopathy. CMR is a gold standard clinical technique to measure cardiac volumes and function, so these data have significant clinical utility. The tables of results include all RA 1D and 2D parameters and volume, and are divided into males/females or all subjects, and in age deciles, when appropriate, or all ages, in order to have applicability for comparison with any other future research data set. For all ages and genders, a volume of 139 mL (75 mL/m^2^) was obtained as the upper limit of normality. With regard to areas, the upper limits of normality were 30 cm^2^ (15 cm^2^/m^2^) in the four chamber view, and 29 cm^2^ (16 cm^2^/m^2^) in the 2-chamber. The upper limits of normality for diameters in the 4-chamber and 2-chamber views were longitudinal 6.6 cm (3.6 cm/m^2^) and 6.5 cm (3.5 cm/m^2^), and transverse 5.8 cm (3.2 cm/m^2^) and 5.7 cm (3.2 cm/m^2^), respectively.

RA volume measurements with CMR have been validated in the past using an excised heart cast model [21], but there is little peer-reviewed validated literature on RA reference dimensions to compare our data [12,22,23], with very different results that are partly due to differences in imaging sequences, imaging views, acquisition, analysis and characteristics of the patients included. Some authors have published reference ranges for RA dimensions with CMR but we have not found any other study in which all 1D, 2D and 3D parameters were measured with CMR. Anderson et al. [13], measured maximal RA area and depth in end-systole in the four-chamber view and obtained an upper limit of normality of 23.5 cm^2^ for area and 55.6 mm for depth, with no significant differences between men and women (4-chamber RA area 22.5 vs 18.8 cm2, RA depth 52.7 vs 51.5 mm). For the sake of simplicity, these authors concluded that an area <24 cm2 and a depth <58 mm included the upper 95th percentile of the normal range both for the left and right atria and best separated cardiomyopathic from normal hearts, these values are slightly lower than ours. Sievers et al. [11], measured RA volumes in 70 subjects using the short axis method and published an upper limit of normality of RA volume of 170.4 mL (89.2 mL/m2), which is higher than our results. We think that this difference in the upper limit of normality is attributable to a different, more heterogeneous, subject population, as both the imaging sequence, acquisition method- with retrospective gating- and image analysis were similar to ours. In fact the mean RA volume in Sievers’s study is 101 mL with a standard deviation of 30.2 mL, while we found a very similar RA volume, of 100 mL, with a lower standard deviation, 20 mL, and thus a narrower normal range and lower upper limit.

We observed that nearly all non-indexed 1D, 2D and 3D parameters were significantly higher in males, while these differences disappeared in most indexed parameters. This is in accordance with the findings by Sievers et al. [11], who observed higher absolute volumes in males but no differences after adjusting by body surface area.

With respect to age, we found no differences in RA volume with increasing age in this group of healthy individuals, and only found significant differences in both absolute and indexed transverse diameter and area in the 2-chamber view. The RA is exposed to right ventricular diastolic pressure and, because of its thin walls, tends to dilate when pressure increases. In a healthy population this is not the case and, though with increasing age a mild degree of myocardial stiffness could be expected, no significant effect on RA volume was observed. Diastolic function parameters derived from ventricular time-volume curves in this healthy population have been published elsewhere. This has been corroborated in a post-mortem study [19], and in a number of *in-vivo* studies with different imaging techniques. Sievers et al. [11], observed no age related differences in RA volume with CMR. Aune et al. [24], measured RA volume by 3D echocardiography in 166 healthy subjects and found that normal aging does not increase RA size. On the other hand Grapsa et al. [20], studied 62 consecutive patients with pulmonary arterial hypertension and observed increased RA sphericity index, which was a good predictor of clinical outcome.

### Comparison with echocardiographic studies and other imaging techniques

CMR does not require geometric assumptions to measure atrial volume, so volumes obtained with retrospectively gated CMR are likely to differ significantly from those obtained with 1D and 2D echocardiography. Echocardiographic reference values have been quoted as 4.2 ± 0.4 cm for RA depth and 14.0 ± 1.5 cm^2^ for RA area [25], lower than our measurements. Differences are also related to the greater precision of CMR compared with echo, the improved spatial resolution of endocardial border and slightly different anatomic views. Similarly Wang et al. [26], estimated with RA volume with the echocardiographic area length method from the apical four-chamber view and obtained an upper limit of normality of 31 mL/m^2^, far below our values, which can be explained by the different methodological approach and technical equipment. Whitlock et al. [27] compared RA volume estimated using the echocardiographic area-length method and CMR and found that echocardiography caused a significant underestimation of RA volume. Currently, 3D echo is a more reproducible and robust method for measuring RA volume. Aune et al. [24], obtained with 3D echo an upper normal value of 47 mL/m^2^ for the entire group, with a higher upper reference value for males (50 mL/m^2^) than females (41 mL/m^2^), still lower than our results. Noteworthy, in this 3D echo study RA volume was found to be 15% higher than normal left atrial volume, similar to our findings comparing to our previous paper on LA volumes, and no significant correlation was found between RA volume and age. Keller et al. [23], validated echo derived RA volumes against CMR and found an excellent correlation for 3D echo derived RA volume (r = 0.91), with a significant underestimation of 12.06 mL, and worse correlation for 2D echo using single 4-chamber summation of disks algorithm (r = 0.79). This underestimation could be due to a number of reasons including the higher spatial resolution of CMR, which permits more accurate border detection and better delineation of volumes within the trabeculae, low lateral resolution of the ultrasound beam, the gain dependent nature of the boundary echoes, and the lower temporal resolution of 3D echo and reconstruction algorithms. These authors also suggested that CMR may overestimate RA volume by including the cava venous confluence, the appendage volume and the annular plane, in our study we included the atrial appendage but carefully excluded the cava veins, and as for the tricuspid annular plane this was carefully delineated in the end-systolic phase.

Cardiac computed tomography (CCT) has also been used to measure RA volume, with reference values higher than ours. Lin et al. [28], measured RA volume with 64-row CCT in 103 healthy normotensive non-obese volunteers and obtained a reference value of 111.9 ±29 mL with a reference range of 54.9-168.9 mL. This difference compared to our results could be at least in part explained by differences in the recruited subjects, as this was not a population study, 57% of subjects were male and slightly older. On the contrary, Takahashi [29] measured atrial volume with 320-slice computed tomography and semi-automated 3 dimensional segmentation technique and found a normal value of 82.1 ± 44.1 mL, which is smaller than our results, though in this study only 22 subjects were included and thus it is difficult to compare with our study.

### Predictors of RA enlargement and estimators of RA volume

Measurement of RA volume is desirable but may be time-consuming for daily clinical practice. Therefore, 1D and 2D parameters might be a valuable tool to assess RA size. The best independent indicators of RA enlargement in our study were an area >16 cm^2^/m^2^ and a longitudinal diameter >3.5 cm/m^2^ in the 2-chamber view. In the study by Anderson et al. [13], a non-indexed RA area <24 cm^2^ and depth <5.8 cm were the parameters that best distinguished normal from abnormal atria. We have not found any other study with which to compare our data.

With respect to RA volume estimators, we found that the best method included measurement of area in the 2 and 4-chamber views. We correlated real volume with estimated volumes derived from this method and from the traditionally used single plane area-length method. Both correlated well but caused a significant underestimation of RA volume, with worse accuracy for the area-length method (mean difference of 22.7 ±19.8 mL). Some studies have also compared methods of volume estimation with real volumes, with different results. Sievers et al. [11], also compared the single plane area-length method with the short axis method in 70 healthy subjects and found that the former overestimated RA volume.

## Conclusions

Right atrial dimensions do not vary with gender after adjustment for body surface area and only a few show differences with age. References ranges are supplied with this report in both tabular and graphical form and are of significant clinical and research utility for the interpretation of CMR studies. Also, best predictors of RA enlargement are provided.

## Abbreviations

(RA): Right atrial; (CMR): Cardiovascular magnetic resonance; (SSFP): Steady state free precession; (AV): Atrioventricular.

Professor Pennell is a consultant to Siemens and a director of Cardiovascular Imaging Solutions. The other authors have no conflicts to declare. This research was supported by CORDA and the British Heart Foundation. Research support was also received from Siemens.

## Competing interests

The authors declare that they have no competing interests.

## Authors’ contributions

AMM- Study design, subject scanning, data selection, data processing, writing manuscript. JCS- Subject recruitment, data selection, data processing. MR- Study design, statistical analysis. SKP- Study design, subject scanning. DJP- Study design, editing manuscript. All authors read and approved the final manuscript.
